# Reiterative ventricular fibrillation caused by R-on-T during temporary epicardial pacing: a case report

**DOI:** 10.1186/s40981-016-0029-6

**Published:** 2016-01-15

**Authors:** Yuki Nakamori, Takuma Maeda, Yoshihiko Ohnishi

**Affiliations:** Department of Anesthesiology, National Cerebral and Cardiovascular Center, 5-7-1 Fujishirodai, Suita, Osaka 565-8565 Japan

**Keywords:** Cardiac pacemaker, Artificial, Epicardial temporary pacing, R-on-T phenomenon, Undersensing, Slew rate, Premature ventricular contraction

## Abstract

Epicardial pacemaker wire insertion is standard following cardiothoracic surgery. However, undersensing of pacing wires may cause the R-on-T phenomenon, which induces ventricular fibrillation. We report a case of a male patient with severe mitral regurgitation scheduled for mitral valve replacement who experienced two ventricular fibrillation episodes related to the R-on-T phenomenon caused by undersensing of the epicardial pacing wire. Both undersensing events happened despite an appropriately low sensing threshold. Notably, the stimulated T wave followed the QRS of the premature ventricular contraction (PVC). This case suggests that a PVC’s R wave may be undersensed despite a low sensing threshold. This critical complication may have occurred because pacemakers sense R waves using a slew rate, which is the quotient of voltage over time. As a result, pacemakers may undersense wide QRS waves such as PVCs. Avoiding this dangerous phenomenon completely is not possible using epicardial pacemakers; therefore we recommend carefully adapting epicardial pacing especially when PVC waves occur frequently.

## Background

Epicardial pacemaker wire insertion is standard following cardiothoracic surgery in most centers [1]. These wires are valuable in diagnosing and treating cardiac arrhythmias following surgery, maintaining an appropriate heart rate, and may facilitate the reestablishment of circulatory integrity and normal hemodynamics [2]. They also suppress both atrial and ventricular tachyarrhythmias [3]. The R-on-T phenomenon is a well-known entity that predisposes to dangerous arrhythmias, including ventricular fibrillation (Vf), a fatal arrhythmia. The phenomenon is also related to undersensing of temporary pacing wires. To check its sensitivity, the pacemaker rate should be set below the endogenous rate (if present), and placed in ventricular demand (VVI), atrial demand (AAI), or atrioventricular universal (also known as dual-chamber (DDD)) modes. The sensitivity is increased until the sense indicator stops flashing and that number is the threshold. It is recommended to leave the pacing generator set at half the pacing threshold. If there is no endogenous rhythm, it is impossible to determine the pacemaker sensitivity, in which case the sensitivity is typically set to 2 mV [4]. There is currently no consensus on the threshold for premature ventricular contraction (PVC).

Previous reports have presented electrocardiography (ECG) in cases of undersensing in temporary pacing wires [1, 5]; however, the cause is unclear. The R-on-T pacing because of undersensing of temporary pacing wires is an unavoidable and critical problem for most patients undergoing surgery using cardio-pulmonary bypass (CPB). Anesthesiologists must understand the mechanism of undersensing to provide better pacemaker management for patients undergoing cardiac surgery using CPB to protect their safety.

We herein report two Vf episodes in a single patient related to the R-on-T phenomenon caused by undersensing of the pacing wire. In this case, undersensing occurred despite a sufficiently low threshold, on which the T wave followed the QRS of the PVC, which is an important finding for anesthesiologists who manage epicardial pacemakers.

## Case presentation

A 65-year-old male with severe mitral regurgitation and moderate tricuspid regurgitation was scheduled for mitral valve replacement, tricuspid annuloplasty, and maze surgery. The degree of heart failure was New York Heart Association class II and atrial fibrillation had been present for 18 months. There was no history of syncope or sudden death among the patient’s family members. Preoperative coronary angiography showed no significant stenosis; preoperative ECG revealed a heart rate of 65 bpm and atrial fibrillation. The QRS was narrow without evidence of bundle branch block, and there was no sign of QT prolongation.

**Fig. 1:**
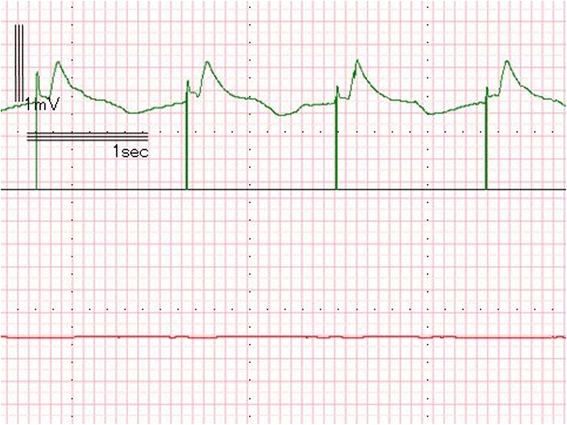
ECG and pressure waveform at 50 bpm VVI pacing. Each VVI pacing spike captures a QRS wave

**Fig. 2:**
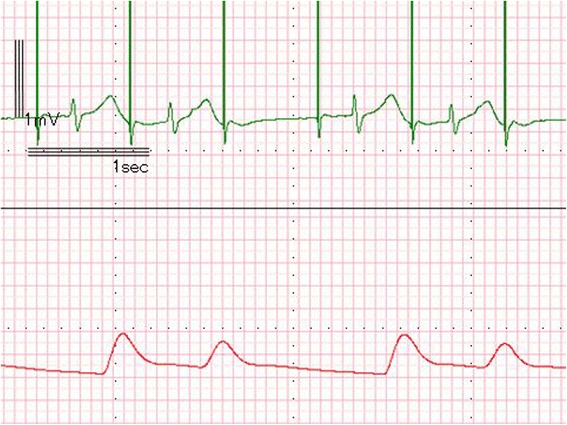
ECG and pressure waveform showing Mobitz type AV block. Each AOO pacing spike captures a P wave, but every third P wave does not induce a QRS wave

General anesthesia was induced with midazolam, fentanyl, and rocuronium and maintained by continuous injection of propofol, remifentanil, and rocuronium. The operation proceeded without problems and his mitral valve was replaced with a biological valve. The left atrium vent and aortic root cannula were removed after using transesophageal echocardiography (TEE) to confirm that the intracardiac air had been completely removed. We then attempted to withdraw from CPB and began VVI pacing at 50 bpm (Fig. 1). The CPB time was 185 min and the cardiac arrest time was 155 min. After 30 min, we attempted atrial asynchronous (AOO) pacing, but the patient’s atrioventricular (AV) node did not completely conduct supraventricular electrical activity and the ECG showed Mobitz type AV block (Fig. 2); therefore, we elected to maintain VVI pacing. Before sternal closure, epicardial pacemaker wires were inserted on the anterior right ventricle (RV) and the anterior right atrium and we changed the direct grasping myocardial lead to epicardial pacemaker wires. Because the patient’s endogenous ventricular rhythm was only 30 bpm immediately post-operatively, we chose VVI pacing at 90 bpm. At that time, both the electrolyte levels and acid–base balance were within the allowable ranges; Na, 143 mEq/L; K, 4.4 mEq/L; Cl, 116 mEq/L; and standard base excess, −3.1 mmol/L. Additionally, the patient’s rectal temperature was kept above 36.2 °C after weaning from CPB.

**Fig. 3:**
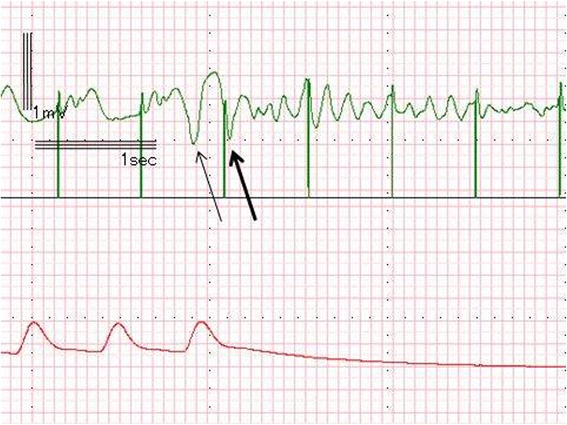
ECG and pressure waveform during the first episode of ventricular fibrillation. This ECG shows the R-on-T phenomenon resulting from undersensing of a PVC. A pacing spike hit the T wave (large arrow) following a PVC (small arrow)

**Fig. 4:**
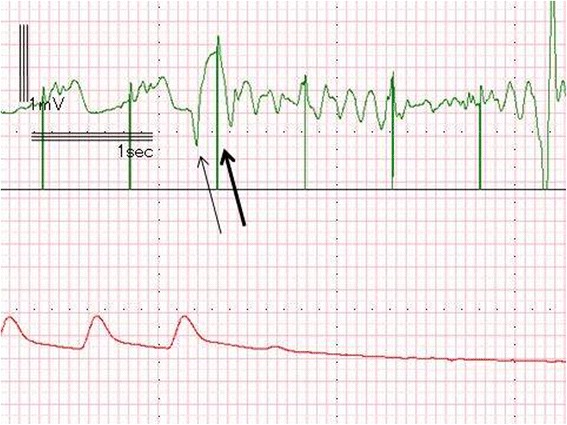
ECG and pressure waveform during the second episode of ventricular fibrillation. This ECG also shows the R-on-T phenomenon resulting from undersensing of a PVC. A pacing spike hit the T wave (large arrow) following a PVC (small arrow) in this episode, as well

During transfer from the operation room to the intensive care unit (ICU), the ECG showed Vf (Fig. 3). External defibrillation with 270 J was administered immediately, which resulted in recovery of the electrical rhythm; ventricular pacing was then able to capture ventricular contraction. We assumed that undersensing had occurred; therefore, the ventricular sensing threshold was changed from 10 mV to 2 mV, which is a sufficiently low sense threshold, recommended in cases where there is no endogenous rhythm [4]. However, within only two minutes of the first Vf, the ECG again showed a Vf waveform (Fig. 4). The second Vf was also treated by external defibrillation. After these two Vf episodes, we performed blood gas analysis and again obtained values within the allowable ranges: Na, 144 mEq/L; K, 4.6 mEq/L; Cl, 117 mEq/L; and standard base excess, −3.8 mmol/L.

We subsequently evaluated the electronically recorded ECG waveforms collected during anesthesia and found that the Vf episodes were induced by the R-on-T pacing. Neither T wave during the Vf episodes followed the pacing-induced QRS complex, but instead followed the T wave of the premature ventricular contractions.

To prevent further the R-on-T pacing, the pacemaker was set at AOO pacing at 70 bpm during the ICU stay. Because of the presence of the AV block, AOO pacing could not produce enough subsequent ventricular contraction; therefore, we used norepinephrine to maintain the body pressure at first. However, the patient’s AV conduction recovered over time, and he was free from temporary epicardial pacing and any catecholamines or vasopressors support on postoperative day 1. He was discharged from the ICU on postoperative day 5 and finally discharged from the hospital without any neurological sequelae on postoperative day 13.

## Discussion

This was a case of the R-on-T phenomenon caused by undersensing of the pacing wire in a single patient. Notably, the T wave followed the QRS of the PVC. Understanding this phenomenon requires the answers to two questions: First, why were the pacing spikes not inhibited? Second, what caused the PVCs?

The R-on-T pacing in this patient occurred despite lowering the sense threshold to 2 mV, suggesting that simple undersensing is unlikely to explain this phenomenon. Lowering the sense threshold even further than in this case is not a reasonable management in temporary epicardial pacing for patients with prolonged AV delay. Like this patient, severe AV block cases require ventricular pacing to maintain sufficient cardiac output because their ventricular contractions are too rare to do so; therefore excessively low sensing thresholds are not an option. Low threshold sensing numbers cause the pacemaker to sense external factors such as electrical scalpel stimulation or postural change. If pacing is excessively inhibited, patients suffer critical bradycardia and low-output syndrome.

Dispersion of the duration of the action potential and electrical inhomogeneity in the myocardium may explain the undersensing [6]. The electrical voltage waveforms that pacemakers detect are not equal to those identified by surface electrocardiography. Surface electrocardiography reflects electrical activity of the entire heart while the pacemaker electrode contacts the external cardiac muscle directly and senses only the electrical excitation of surrounding cells. Surface electrocardiography is related to space and epicardial wires are related to linear or punctual factors. With peri-lead fibrous or necrotic tissue, pacemakers cannot detect depolarization despite QRS waves appearing on the ECG monitor. Also, the literature provides little information on the best locations for epicardial pacing. In the presented case, the temporary epicardial electrode was placed on the anterior RV, which is the most widely used location for wire implantation because of the easy access. However, one study reported that the anterior RV location provides sub-optimal sensing and stimulation thresholds [7].

In the presented case, the undersensed QRS wave was a wide PVC; therefore, QRS width could be a contributing factor. If the device recognizes the R wave by slew rate, which is the quotient of voltage over time, a wide QRS may impair accurate sensing of R voltage. The duration of the QRS wave would be longer and the slew rate would be smaller despite having the same R wave voltage. Therefore, temporary epicardial pacing devices set at a clinically valid threshold may not detect abnormally wide QRS waves without omission.

We provided anesthetic management to prevent arrhythmias in this patient. We maintained intravascular volume, preload, afterload, heart rate, and contractile force within normal limits by referring to TEE, arterial blood pressure, pulmonary arterial pressure, central venous pressure, cardiac output, and mixed venous blood saturation in oxygen by Swan-Ganz Catheter. We did not use excessive amounts of catecholamines and only 3 mcg/kg/min dopamine was injected. We also maintained the patient’s electrolytes and body temperature at normal levels. The ECG ST slope elevated slowly by the time of sternal closure. TEE performed after the two Vf episodes showed poor contraction of the right coronary artery (RCA) perfusion segments, often caused by RCA ischemia. Intracardiac air was removed carefully and there was little probability that coronary spasm or partial pericardial tamponade occurred. Bleeding in or around the epicardial pacing wires tends to occur in the immediate postoperative period and pericardial hematoma formation around epicardial pacing wires has been reported [8]. Myocardial infarction related to the epicardial pacing wire itself has also been reported [9]. The hypothesis that ischemic RCA perfusion induced frequent PVCs is reasonable.

Following this case, we reviewed our anesthetic records from the past 7 years and found one case of Vf resulting from the R-on-T wave phenomenon; however, we concluded that the cause of the R-on-T in that case was an excessively high sensing threshold. Also, the undersensed QRS wave was one following a P wave.

This case was our first experience of Vf caused by “R on PVC T wave” phenomenon. Some authors suggest that routine use of temporary epicardial pacing wires after valve surgery is only necessary for high risk patients [10, 11]. In addition to the R-on-T pacing, major complications of epicardial wires include infection, myocardial damage, perforation, tamponade, and disruption of coronary anastomoses [4]. Insertion can prolong the operative time and increase the risk of bleeding and patients are also at risk for ventricular arrhythmias during removal of epicardial pacing wires [12]. Therefore, it is important to consider the risks of using temporary epicardial pacing.

## Conclusions

We report a case of Vf episodes related to the R-on-T phenomenon caused by undersensing of a QRS wave despite an appropriately low sensing threshold. Temporary epicardial pacing is very effective for bradycardia or low output syndrome after CPB but there are instances in which a PVC’s R wave may be undersensed despite an appropriately low sensing threshold. This undersensing can cause the R-on-T phenomenon, which may induce critical arrhythmia. It is very important to assess the use of temporary epicardial pacing with high-frequency PVC waves.

## Consent

Written informed consent was obtained from the patient for publication of this Case report and any accompanying images. A copy of the written consent is available for review by the Editor-in-Chief of this journal.

## Abbreviations

AAI: atrial demand
AOO: atrial asynchronous
AV: atrioventricular
CPB: cardio-pulmonary bypass
DDD: dual-chamber
ECG: electrocardiography
ICU: intensive care unit
PVC: premature ventricular contraction
RCA: right coronary artery
RV: right ventricle
TEE: transesophageal echocardiography
Vf: ventricular fibrillation
VVI: ventricular demand
